# Genetic Associations with Diabetes: Meta-Analyses of 10 Candidate Polymorphisms

**DOI:** 10.1371/journal.pone.0070301

**Published:** 2013-07-29

**Authors:** Linlin Tang, Lingyan Wang, Qi Liao, Qinwen Wang, Leiting Xu, Shizhong Bu, Yi Huang, Cheng Zhang, Huadan Ye, Xuting Xu, Qiong Liu, Meng Ye, Yifeng Mai, Shiwei Duan

**Affiliations:** 1 Zhejiang Provincial Key Laboratory of Pathophysiology, School of Medicine, Ningbo University, Ningbo, Zhejiang, China; 2 The Affiliated Hospital, Ningbo University, Ningbo, Zhejiang, China; 3 Bank of Blood Products, Ningbo No.2 Hospital, Ningbo, Zhejiang, China; Sapienza, University, Italy

## Abstract

**Aims:**

The goal of our study is to investigate the combined contribution of 10 genetic variants to diabetes susceptibility.

**Methods:**

Bibliographic databases were searched from 1970 to Dec 2012 for studies that reported on genetic association study of diabetes. After a comprehensive filtering procedure, 10 candidate gene variants with informative genotype information were collected for the current meta-anlayses. Using the REVMAN software, odds ratios (ORs) with 95% confidence intervals (CIs) were calculated to evaluate the combined contribution of the selected genetic variants to diabetes.

**Results:**

A total of 37 articles among 37,033 cases and 54,716 controls were involved in the present meta-analyses of 10 genetic variants. Three variants were found to be significantly associated with type 1 diabetes (T1D): *NLRP1* rs12150220 (OR = 0.71, 95% CI = 0.55–0.92, P = 0.01), *IL2RA* rs11594656 (OR = 0.86, 95% CI = 0.82–0.91, P<0.00001), and *CLEC16A* rs725613 (OR = 0.71, 95% CI = 0.55–0.92, P = 0.01). *APOA5* −1131T/C polymorphism was shown to be significantly associated with of type 2 diabetes (T2D, OR = 1.27, 95% CI = 1.03–1.57, P = 0.03). No association with diabetes was showed in the meta-analyses of other six genetic variants, including *SLC2A10* rs2335491, *ATF6* rs2070150, *KLF11* rs35927125, *CASQ1* rs2275703, *GNB3* C825T, and *IL12B* 1188A/C.

**Conclusion:**

Our results demonstrated that *IL2RA* rs11594656 and *CLEC16A* rs725613 are protective factors of T1D, while *NLRP1* rs12150220 and *APOA5* −1131T/C are risky factors of T1D and T2D, respectively.

## Introduction

The prevalence of diabetes is soaring up in the recent decades. The global number of diabetes patients was 173 million in 2002 and will increase to 350 million by 2030. As a group of metabolic diseases characterized with high blood sugar, most diabetes is caused by either a lack of insulin for type 1 diabetes (T1D) or a blockage in the insulin signaling pathway for type 2 diabetes (T2D). The classical symptoms of diabetes consist of polyuria, polydipsia, polyphagia and weight loss. Diabetes also causes damages to blood vessels and capillaries that may eventually lead to coronary heart diseases and blindness, respectively.

T1D and T2D are two major types of diabetes [1]. Microbial exposures and sex hormones together with lifestyle factors have been shown to be important to the development of this complex disease [2], [3]. Besides environmental factors, twin studies have demonstrated a strong heritability for diabetes [4], [5] and insulin related phenotypes [6]–[8]. A handful of candidate genes have been found for both the risk and complex traits of the two major types of diabetes [3], [9]–[12].

T1D is an autoimmune disease. Little or no insulin is produced by pancreatic beta cells that may be mistakenly attacked after an infection or some other triggers. The present meta-analyses of T1D focus on four immunomodulatory genes including *IL2RA*, *NLRP1*, *IL12B* and *CLEC16A. IL2RA* gene encodes the α-chain of IL-2 receptor (IL-2R) complex which acts as an important modulator to regulate T-cell immune response [13]. *NLRP1* gene encodes a member of the Ced-4 family of apoptosis proteins that can stimulate innate immunity [14]. *IL12B* gene encodes a subunit of an important immunomodulatory cytokine, IL-12. IL-12 induces production from NK and T cells of interferon γ (IFN-γ) which favors Th1 cell differentiation [15]. *CLEC16A* encodes C-type lectin domain family 16 (CLEC16A) protein highly expressed on B-lymphocytes, natural killer (NK) and dendritic cells [16].

The impairment of insulin signaling in T2D is complex. Insulin signaling is involved in both glucose and lipid metabolism. In the present meta-analyses of T2D, we selected 2 genes in glucose metabolism (*SLC2A10* and *CASQ1*), 2 genes in lipid metabolism (*APOA5* and *KLF11*), and 2 genes in signal transduction (*ATF6* and *GNB3*). *SLC2A10* gene encodes a member of the facilitative glucose transporter family with an effect on maintaining glucose homeostasis. *CASQ1* gene encodes acidic glycoprotein calsequestrin 1 (CASQ1) that is a calcium storage protein and calcium is considered to regulate the expression of the insulin-responsive glucose transporter GLUT4 [17]. *APOA5* is located on human chromosome 11q23, in the APOA1/APOC3/APOA4 gene cluster [18]. *KLF11* encodes Kruppel-like factor 11 with the function of regulating hepatic lipid metabolism [19]. *ATF6* encodes UPR transducer unfolded protein that is related to the endoplasmic reticulum stress in the β-cell pathogenesis of type 2 diabetes [20]. *GNB3* encodes the β3 subunit of hetero-trimeric G proteins in insulin signaling [21].

Associations between single-nucleotide polymorphisms (SNPs) of the above 10 genes and diabetes (including T1D and T2D) have been reported in different ethnic populations [16], [22]–[57]. Here we performed a series of meta-analyses for these SNPs whose allelic frequencies are often substantially different among multiple ethnic populations. The goal of our study is to evaluate the overall contribution of these SNPs to diabetes susceptibility in combined populations using a meta-analysis approach.

## Materials and Methods

### Search Strategy and Study Selection

An initial search was performed through online databases including PubMed, Embase, SpingerLink, Web of Science, Chinese National Knowledge Infrastructure (CNKI), and Wanfang. The keywords comprise the terms including “diabetes” together with “SNP” or “polymorphism” or “variants” or “mutation”. The selection of studies in our meta-analysis was abided by the criteria as follows: (1) case-control studies; (2) selected studies have sufficient data to calculate ORs with the corresponding 95% CIs; (3) every polymorphism has at least 3 independent datasets from the retrieved articles; (4) selected polymorphisms have not been addressed in previous meta-analysis of diabetes. Finally, the current meta-analysis involved a total of 10 genetic variants comprising *NLRP1* rs12150220, *IL2RA* rs11594656, *CLEC16A* rs725613,*APOA5* −1131T/C, *SLC2A10* rs2335491, *ATF6* rs2070150, *KLF11* rs35927125, *CASQ1* rs2275703, *GNB3* C825T, and *IL12B* 1188A/C.

### Statistical Analysis

All the analyses were performed in Review Manager (version 5.0, The Cochrance Collaboration [58]). The combined ORs and the corresponding 95% CIs were calculated and demonstrated in the forest plots using the fixed or the random effects model. Heterogeneity was measured in our meta-analysis using Cochran’s Q and the inconsistency index (I^2^) statistic [59]. Funnel plots were used to detect whether there were obvious publication bias among the involved studies. An I^2^ value of equal to or greater than 50% indicates a substantial heterogeneity among the studies in the meta-analysis that used a random-effect model for the analysis. For I^2^ value less than 50%, a fixed-effect model will be applied for the meta-analysis. The combined ORs and the corresponding 95% CIs were calculated using the fixed-effect model or the random-effect model if I^2^ is less than 50%. P values less than 0.05 were considered to be significant.

## Results

**Figure 1:**
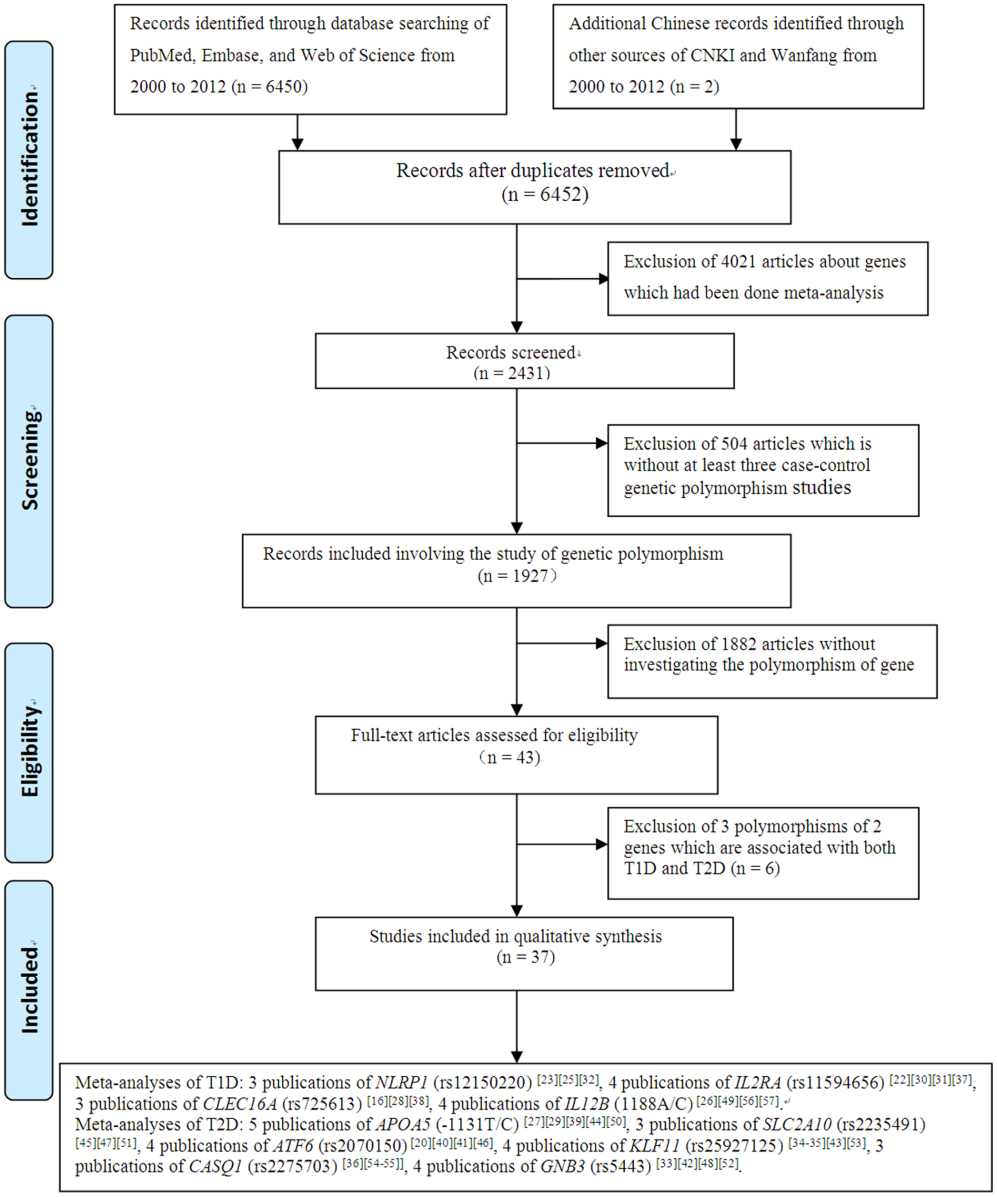
Flow diagram of selecting studies for meta-analysis.

As shown in Figure 1, our initial search for the genetic studies of diabetes retrieved 6,452 articles from PubMed, Embase, Web of Science, CNKI and Wanfang from 2000 to 2012. Among them, 4,021 studies were involved with genes reported in previous meta-analyses and thus discarded for further analysis. A total of 504 articles were again filtered out because they failed to accumulate at least three independent genotypic datasets for the same genetic variants. Among the rest 1,927 studies, 1,882 studies with unconcerned SNPs were removed. At last, there were 42 case-control studies from 37 articles (including 35 articles in English and 2 in Chinese) for the current meta-analyses. There were four SNPs of T1D (Table 1) and six SNPs of T2D (Table 2) involved in our present study.

**Table 1:** Characteristics of individual T1D studies in the meta-analyses.

Gene	SNP	Year	Author	Ethnic Group	Case/Control (n)	Allele 1 (Case/Control)	Allele 2 (Case/Control)
*IL2RA*	rs11594656					T	A
		2007	Christopher E Lowe	Caucasian	2874/2484	4482/3726	1266/1242
		2007	Christopher E Lowe	Caucasian	5259/6809	8199/10248	2319/3370
		2008	Deborah J Smyth	Caucasian	8064/9339	12548/14083	3580/4595
		2009	Eiji Kawasaki	Japanese	881/606	1715/1175	47/37
		2012	M. Fichna	Caucasian	445/671	701/994	189/348
*NLRP1*	rs12150220					T	A
		2009	NF Magitta	Caucasian	1067/3177	929/2987	1205/3367
		2010	A. PONTILLO	Brazilian	196/192	248/255	144/129
		2011	Magdalena Zurawek	Caucasian	221/254	230/270	212/238
*IL12B*	1188 A/C					A	C
		2002	Lorenza Nistico	Caucasian	470/544	662/787	278/301
		2002	RM McCormack	Caucasian	120/330	194/533	46/127
		2005	José L. Santiago	Caucasian	300/516	453/773	147/259
		2010	A.E.Altinova	Turks	91/87	133/120	49/54
*CLEC16A*	rs725613					A	C
		2007	Hakon Hakonarson	Caucasian	561/1143	785/1395	337/891
		2009	M Zoledziewska	Caucasian	1037/1706	969/1473	1105/1939
		2009	Xiao pan Wu	Chinese	205/422	352/643	58/201

**Table 2:** Characteristics of individual T2D studies in the meta-analyses.

Gene	SNP	Year	Author	Ethnic Group	Case/Control (n)	Allele 1 (Case/Control)	Allele 2 (Case/Control)
*APOA5*	−1131 T/C					T	C
		2005	Sheng kai Yan	Chinese	172/155	231/224	113/86
		2006	P. J. Talmud	Caucasian	142/2438	273/4401	11/295
		2007	Guang hua Zhai	Chinese	71/152	81/214	61/90
		2008	Xue feng Li	Chinese	256/340	322/468	190/212
		2008	Yan Qiao	Chinese	154/206	222/313	86/99
*SLC2A10*	rs2235491					G	A
		2005	Karen L. Mohlke	Caucasian	784/401	1476/746	92/56
		2005	Jennifer L Bento	Caucasian	296/305	568/592	24/18
		2006	W. H. Lin	Chinese	375/377	691/683	59/71
*ATF6*	rs2070150					C	G
		2006	Farook Thameem	Pima Indian	561/399	913/626	209/172
		2007	Steven J. R. Meex	Caucasian	367/377	670/693	64/61
		2007	Winston S. Chu	Caucasian	191/188	364/350	18/26
		2011	Cheng Hu	Chinese	1892/1808	1181/1088	2603/2528
*KLF11*	rs35927125					A	G
		2005	Bernadette Neve	Caucasian	313/313	562/517	64/109
		2006	Jose C. Florez	Caucasian	469/468	850/854	88/82
		2006	Jose C. Florez	Caucasian	504/503	906/910	102/96
		2006	Jose C. Florez	Canadian	111/109	195/188	27/30
		2006	Jose C. Florez	Caucasian	1207/1198	2129/2103	285/293
		2006	Jose C. Florez	Caucasian	1000/997	1789/1779	211/215
		2008	T. Tanahashi	Japanese	925/893	1850/1786	0/0
		2008	Lijun Ma	Pima Indian	1455/1816	2780/3457	130/175
*CASQ1*	rs2275703					A	C
		2004	Mao Fu	Caucasian	145/358	90/305	200/411
		2004	Swapan Kumar Das	Caucasian	190/119	205/117	175/121
		2007	Thomas Sparsø	Caucasian	1391/4575	1452/4841	1330/4309
*GNB3*	rs5443					C	T
		2005	Jawad G. Kiani	Arab	256/254	178/246	334/262
		2006	G. Andersen	Caucasian	1358/4723	1855/6574	861/2872
		2007	Tetsuo Hayakawa	Japanese	427/388	445/338	409/388
		2008	Makoto Daimon	Japanese	230/2576	259/2740	201/2712

**Figure 2:**
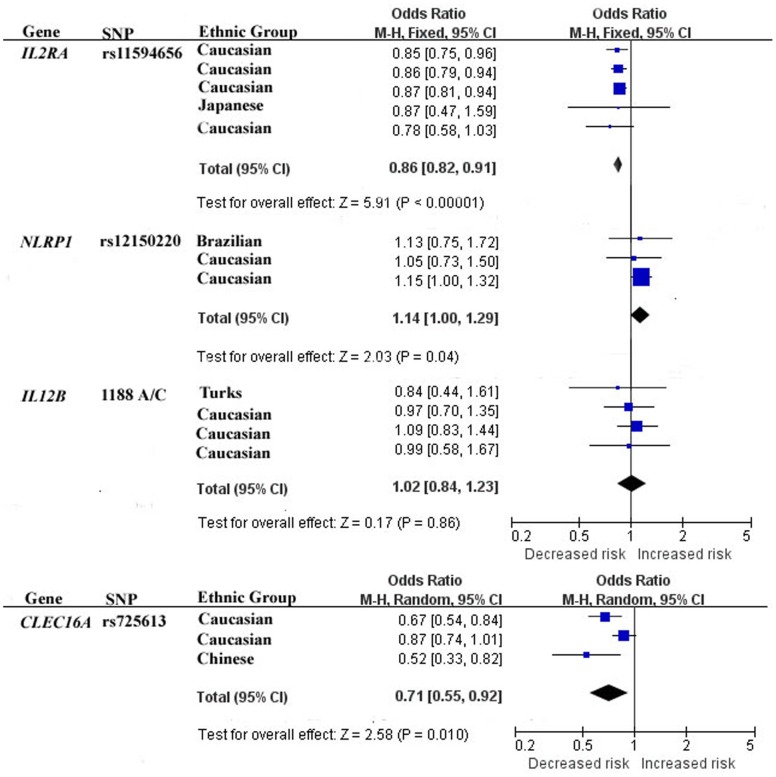
Forest plots of the association studies between four SNPs and T1D.

**Figure 3:**
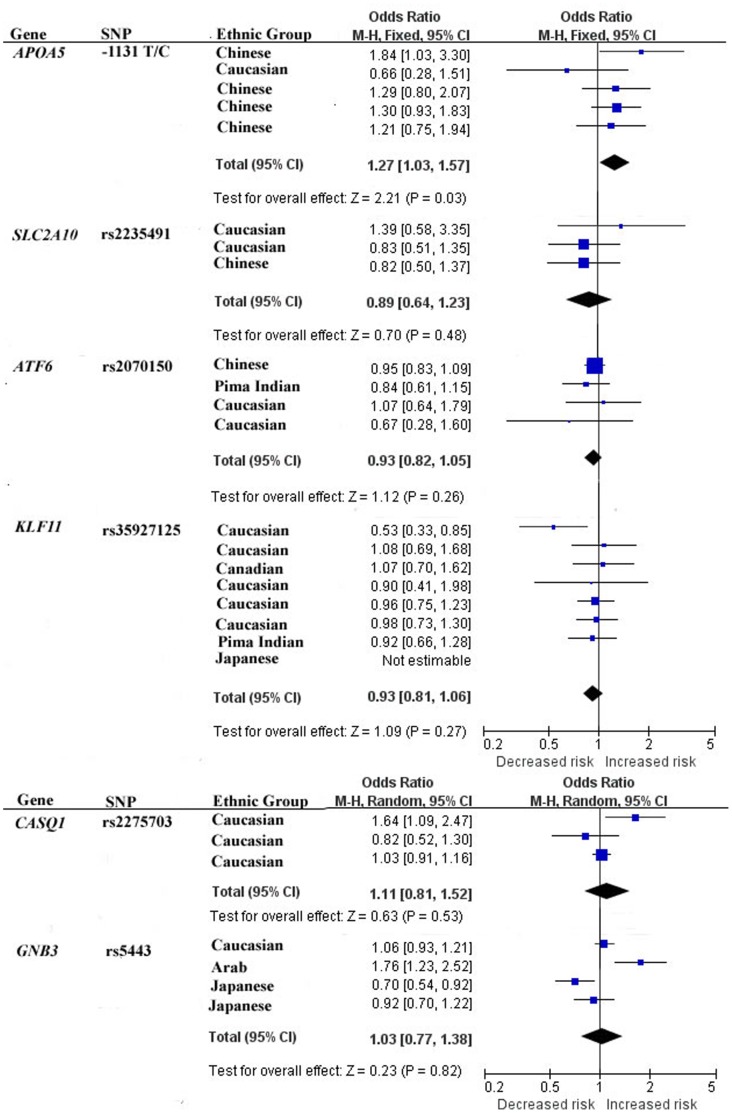
Forest plots of the association studies between six SNPs and T2D.

**Figure 4:**
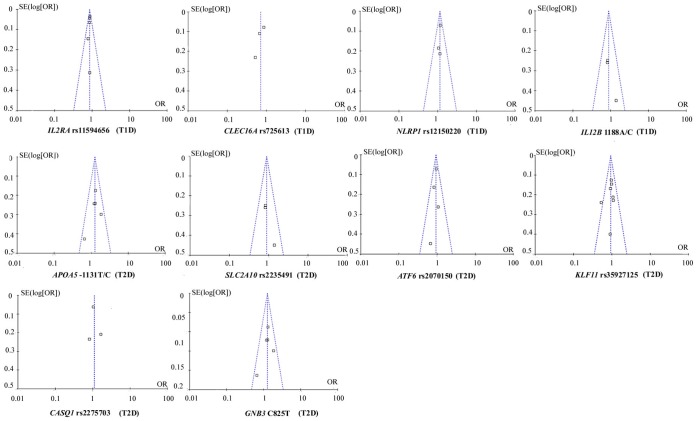
Funnel plots of the studies involved in the 10 meta-analyses.

No evidence of statistical heterogeneity was observed for 7 SNPs (Figures 2 and 3, and Table 3), including rs11594656 of *IL2RA* gene (I^2^ = 0%), rs12150220 of *NLRP1* gene (I^2^ = 0%), 1188A/C of *IL12B* gene (I^2^ = 0%), −1131T/C of *APOA5* gene (I^2^ = 1%), rs2335491 of *SLC2A10* gene (I^2^ = 0%), rs2070150 of *ATF6* (I^2^ = 0%), and rs35927125 of *KLF11* gene (I^2^ = 7%). No visual bias was showed in the meta-analyses of these 7 SNPs (Figure 4 and Table 3). Our data also demonstrated a significant heterogeneity of the rest 3 SNPs that comprise rs725613 of *CLEC16A* gene (I^2^ = 69%), rs2275703 of *CASQ1* gene (I^2^ = 65%), and C825T of *GNB3* gene (I^2^ = 82%). Therefore random-effect tests were applied for the meta-analyses of the above 3 SNPs. Their funnel plots were demonstrated in Figure 4 and no visual bias was observed for the 3 meta-analyses.

**Table 3:** Additional characteristics of individual T1D and T2D studies in the meta-analyses.

T1D	Gene	SNP	Model	Heterogeneity index	P values
	*IL2RA*	rs11594656	Fixed effectmodel	0%	<0.00001
	*NLRP1*	rs12150220	Fixed effectmodel	0%	0.04
	*IL12B*	1188 A/C	Fixed effectmodel	0%	0.86
	*CLEC16A*	rs725613	Random effectmodel	69%	0.01
**T2D**	**Gene**	**SNP**	**Model**	**Heterogeneity index**	**P values**
	*APOA5*	−1131 T/C	Fixed effectmodel	1%	0.03
	*SLC2A10*	rs2235491	Fixed effectmodel	0%	0.48
	*ATF6*	rs2070150	Fixed effectmodel	0%	0.26
	*KLF11*	rs35927125	Fixed effectmodel	7%	0.27
	*CASQ1*	rs2275703	Random effectmodel	65%	0.53
	*GNB3*	rs5443	Random effectmodel	82%	0.82

Meta-analysis of rs12150220 of *NLRP1* gene was involved with 3 studies [23], [25], [32] among 833 T1D cases and 3,623 controls. As shown in Figure 2, our result indicated that rs12150220 of *NLRP1* gene was significantly associated with T1D risk in the Caucasian and Brazilian populations (the overall OR = 0.71, 95% CI = 0.55–0.92, P = 0.01). Meta-analysis of rs11594656 of *IL2RA* gene among 17,523 T1D cases and 19,909 controls [22], [30], [31], [37] indicated that rs11594656 of *IL2RA* gene was significantly associated with T1D risk in the Caucasian and Japanese populations (Figure 2, the overall OR = 0.86, 95% CI = 0.82–0.91, P<0.00001). Meta-analysis of rs725613 of *CLEC16A* gene [16], [28], [38] included 1,803 T1D cases and 3,271 controls. As shown in the Figure 2, there was significant association between rs725613 of *CLEC16A* gene and T1D in Caucasian and Chinese populations (the overall OR = 0.71, 95% CI = 0.55–0.92, P = 0.01). Meta-analysis of −1131T/C of *APOA5* gene [27], [29], [39], [44], [50] among 795 T2D cases and 3210 controls indicated that −1131T/C of *APOA5* gene was associated with T2D in Chinese and Caucasian populations (Figure 3, the overall OR = 1.27, 95% CI = 1.03–1.57, P = 0.03). For the rest 6 SNPs, our meta-analyses were unable to find significant associations of them with T1D or T2D.

## Discussion

In the present study, a comprehensive systematic overview of genetic association studies was performed for the susceptibility of T1D and T2D. We scrutinized all the candidate case-control studies to identify the eligible SNPs with at least three independent datasets. Our meta-analyses of 10 polymorphisms showed significant evidence for 3 T1D-associated SNPs (*NLRP1* rs12150220, *IL2RA* rs11594656, and *CLEC16A* rs725613) and 1 T2D-associated SNP (*APOA5* −1131T/C). Our meta-analyses were unable to find significant associations of the rest 6 SNPs with T1D or T2D. Moreover, power analysis showed that there might be a lack of power for the meta- analysis of *SLC2A10* rs2335491 (1,455 cases and 1,083 controls, 39%) under a moderate risk of diabetes (OR = 1.2). These might partly explain our failure to observe significant results for the meta-analyses of some polymorphisms.

After Bonferroni correction, only the association of SNP rs11594656 with T2D remains significant. However, false discovery rate (FDR) test, a less conservative correction for multiple hypothesis testing, shows that the q values are 5.11E-5 for *IL2RA* rs11594656, 0.051 for *NLRP1* rs12150220, 0.026 for *CLEC16A* rs725613, and 0.051 for *APOA5* −1131T/C. This suggests the robustness of our positive results in the meta-analyses, although we can’t exclude a chance of false positive results for *NLRP1* rs12150220, *CLEC16A* rs725613 and *APOA5* −1131T/C. Sensitivity analysis demonstrated there were no significant differences of four significant genetic variants after exclusion, suggesting that the results of our meta-analyses was robust. In addition, we have performed a comprehensive analysis for the Fst values of the involved SNPs. Our results show there are moderate ethnic differences for *ATF6* rs2070150 (Fst = 0.13), although there are minimal heterogeneity among the involved studies from different ethnic groups (I^2^ = 0). And *KLF11A* rs35927125 is monomorphic in Asians, however, its minor allele frequency in Caucasian populations ranges from 8.8–12.2% (Fst = 0.0232). On the contrary, there were little ethnic difference for *CLEC16A* rs725613 and *GNB3* rs5443 (Fst <0.1), although there exist large heterogeneity in the involved studies (I^2^>60%). For *CASQ1* rs2275703, the heterogeneity might come from the discrepancies of the samples in the case-control studies.

Pancreatic β-cell inflammation and apoptosis plays a pivotal role in the pathogenesis of T1D [60]. As a member of the Ced-4 family of apoptosis proteins, *NLRP1* is an important mediator of programmed cell death [61]. *NLRP1* plays a pivotal role in the pathogenesis of some inflammatory diseases [62], [63]. In the present research, we combined three independent datasets and performed a meta-analysis to evaluate the association between *NLRP1* rs12150220 polymorphism and T1D susceptibility. Although large ethnic differences of allele frequency were found for *NLRP1* rs12150220 (T allele frequency: 47–53.1% in Caucasians versus 66.4% in Brazilians), minimal heterogeneity was observed in the meta-analysis of this polymorphism (I^2^ = 0%). Our results support *NLRP1* rs12150220 as a protective genetic factor of T1D and provide hints to clarify the mechanistic role of *NLRP1* gene in the pathogenesis of T1D.

Evidence from both genetic and animal model studies has shown a crucial role of IL-2/IL-2RA in the pathogenesis of T1D [37], [64]–[67]. IL-2/IL-2RA regulates CD4^+^CD25^+^ regulatory T cells so as to maintain immune homeostasis [67]. *IL-2RA* rs12722495 was shown to contribute to the risk of T1D by lowering IL-2 signaling and diminishing the function of CD4^+^CD25^+^ regulatory T cells [64]. Interestingly, there is a significant association of *IL-2RA* rs11594656 as a protective factor with the risk of T1D in Polish population [22]. These two SNPs were 24.726 kb away and not in the same linkage disequilibrium block. Expansion of CD4^+^CD25^+^FOXP3^+^ regulatory T cells through maternal insulin treatment was shown to reduce the risk of T1D in children [68]. Increased resistance to CD4^+^CD25hi regulatory T cell-mediated suppression was showed in T1D patients [69]. Our meta-analysis established a significant association between *IL-2RA* rs11594656 polymorphism and T1D, although the influence of rs11594656 polymorphism on the regulation of *IL-2RA* gene remains to be unveiled in the future.

*CLEC16A* gene is located in the major histocompatibility complex class II region (16p13), and it encodes a member of the C-type lectin domain containing family. *CLEC16A* gene variants were associated with multiple autoimmune diseases such as T1D [38], [70]–[72]. *CLEC16A* gene variants were shown to be associated with the alternative splicing event in the *CLEC16A* transcription [73]. Our results suggested a significant association between *CLEC16A* rs725613 and T1D among 5,074 samples from Caucasian and Chinese populations (P = 0.01). A significant heterogeneity (I^2^ = 69%) among these ethnic samples warranted a replication in additional populations.

High level of glucose was shown to induce expression of *APOA5* [74], [75] that is an efficient regulator of plasma triglycerides (TGs) by enhancing the catabolism of TG-rich lipoproteins [76] and prohibiting the transportation of TGs [77]. *APOA5* could probably play a role in the pathogenesis of T2D by regulating the cholesterol homeostasis [44], [76]. *APOA5* gene variants were also reported to be associated with the lipid levels [50], [78], [79] and the risk of coronary heart disease [80], [81] in T2D patients. Since both environmental factors [82], [83] and other genes [84] interact with *APOA5* gene, our significant observation for *APOA5* −1131T/C polymorphism may only partly explain the risk of T2D. Minimal heterogeneity among the involved studies in our meta-analysis (I^2^ = 1%), however, along with previous results [85], [86], in which ethnic differences were observed for the T allele frequency of *APOA5* −1131T/C among the studies in our meta-analysis (68.8–76% in Chinese versus 93.7% in Caucasians).

There are some limitations in the present study. Firstly, publishing bias might exist in this meta-analysis. Case-control studies with a lack of significant results were much harder to be published than those with positive findings. In addition, only publications in English and Chinese were included in the current meta-analyses. All these may distort the results in our meta-analyses. Secondly, some of the involved case-control studies [22]–[24], [26], [28], [31], [34], [35], [37]–[39], [41], [43]–[46], [49], [51]–[57] didn’t provide information on the exclusion of other diseases (such as coronary artery diseases, hypertension, and etc.) during recruitment. Thirdly, the effects of genetic factors on diabetes risk were confounded by other phenotypic parameters such as body mass index. Therefore, case-control studies with better design are warranted to avoid these confounding factors and replicate our findings in future. Fourthly, due to a lack of enough independent datasets, subgroup analysis and meta-regression were not applied to identify differences in effect and sources of heterogeneity. Lastly, our meta-analysis focused on gene loci with at least three independent studies, and this might prevent those gene loci in two large scale case-control studies from being included in the current meta-analysis.

In conclusion, we identify significant associations between 4 SNPs (*NLRP1* rs12150220, *IL2RA* rs11594656, *CLEC16A* rs725613 and *APOA5* −1131T/C) and diabetes. Meta-analysis among 4,456 samples has confirmed that rs12150220 of *NLRP1* gene is a risk factor of T1D in Caucasian and Brazilian populations. Meta-analysis among 37,432 samples has confirmed that rs11594656 of *IL2RA* gene is a risk factor of T1D in Caucasian and Japanese populations. Meta-analysis among 5,074 samples has confirmed that rs725613 of *CLEC16A* gene is a risk factor of T1D in Caucasian and Chinese populations. Another meta-analysis among 4,005 samples indicates that −1131T/C of *APOA5* gene is a risk factor of T1D/T2D in Chinese and Caucasian populations.
